# Co-generation of hydrogen and power/current pulses from supercapacitive MFCs using novel HER iron-based catalysts

**DOI:** 10.1016/j.electacta.2016.10.154

**Published:** 2016-12-01

**Authors:** Carlo Santoro, Francesca Soavi, Catia Arbizzani, Alexey Serov, Sadia Kabir, Kayla Carpenter, Orianna Bretschger, Plamen Atanassov

**Affiliations:** a Department of Chemical and Biological Engineering, Center for Micro-Engineered Materials (CMEM), University of New Mexico, Albuquerque, NM 87131, USA; b Department of Chemistry “Giacomo Ciamician”, Alma Mater Studiorum – Università di Bologna, Via Selmi, 2, 40126 Bologna, Italy; c J. Craig Venter Institute, 4120 Capricorn Lane, La Jolla, CA 92037, USA

**Keywords:** Supercapacitor, microbial fuel cells, hydrogen evolution, bioenergy, PGM-free catalysts

## Abstract

In this work, four different supercapacitive microbial fuel cells (SC-MFCs) with carbon brush as the anode and an air-breathing cathode with Fe-Aminoantipyrine (Fe-AAPyr) as the catalyst have been investigated using galvanostatic discharges. The maximum power (P_max_) obtained was in the range from 1.7 mW to 1.9 mW for each SC-MFC. This in-series connection of four SC-MFCs almost quadrupled P_max_ to an operating voltage of 3025 mV and a P_max_ of 8.1 mW, one of the highest power outputs reported in the literature. An additional electrode (Ad_HER_) connected to the anode of the first SC-MFC and placed in the fourth SC-MFC evolved hydrogen. The hydrogen evolution reaction (HER) taking place at the electrode was studied on Pt and two novel platinum group metal-free (PGM-free) catalysts: Fe-Aminoantipyrine (Fe-AAPyr) and Fe-Mebendazole (Fe-MBZ). The amount of H_2_ produced was estimated using the Faraday law as 0.86 mMd^−1^cm^−2^ (0.132 L day^−1^) for Pt, 0.83 mMd^−1^cm^−2^ (0.127 L day^−1^) for Fe-AAPyr and 0.8 mMd^−1^cm^−2^ (0.123 L day^−1^) for Fe-MBZ. Hydrogen evolution was also detected using gas chromatography. While HER was taking place, galvanostatic discharges were also performed showing simultaneous H_2_ production and pulsed power generation with no need of external power sources.

## Highlights

• Supercapacitive MFC boosted up power/current pulses.
• In-series connection of 4 microbial fuel cells quadrupled voltage and power output.
• Fe-catalysts showed high hydrogen evolution reaction rate in neutral media.
• Co-generation of electricity and hydrogen using SC-MFCs is here demonstrated.

## 1 Introduction

In the last decade, bioelectrochemical systems (BES) have been studied for their applications in bioenergy, resource recovery and biomass degradation [1], [2]. The two most investigated BESs are namely microbial fuel cells (MFCs) [3], [4] and microbial electrolysis cells (MECs) [5], [6]. While MFCs generate electricity from the oxidation of organic waste as a fuel [3], [4], MECs produce hydrogen or other value added products (VAPs) utilizing electricity generated from external sources [5], [6].

MFCs and MECs utilize electroactive bacteria that degrade organic compounds in the electrolyte, releasing the electrons either through mediators or directly to the surface of the electrode (anode) that works as an electron acceptor [7], [8]. In the MFC, electrons liberated from the degradation of electrolyte organics move through the external circuit to the cathode where oxygen is reduced [9], [10], and net current/power is generated. On the other hand, in an MEC, an external power supply is connected in order to achieve the operational potential (≈−1 V vs Ag/AgCl) at which the hydrogen evolution reaction (HER) takes place at the cathode working in neutral media. Consequently, organics are also degraded and H_2_ is produced, but the energy balance is negative in MECs because an external source of energy is needed [11].

The main difference of MFCs and MECs compared to proton exchange membrane fuel cells (PEMFCs) or electrolyzes are: i) the biotic catalyst (electroactive bacteria) at the anode; ii) the cathode directly exposed to the waste (often biotic cathode) iii) ambient working temperatures; iv) neutral media; and v) utilization of complex biomass as anodic fuel [12], [13]. MFCs and MECs still have several technical challenges such as high cost materials and low power densities that must be addressed to be competitive with conventional fuel cells or electrolyzers; however, many of these can be solved through better-suited electrocatalytic materials and operational changes.

One of the main challenges for MECs is the optimization of the H_2_ evolution reaction (HER) that takes place on the cathode. Similar to the oxygen reduction reaction (ORR), catalysts are generally used to significantly decrease the overpotentials and accelerate the reaction in which hydrogen is evolved. In acidic and alkaline conditions, Ir and Pt are known to be the best catalysts for HER [14], [15]. Platinum is the most used and reported catalyst for HER in MEC [16], [17], but the high cost compared to the hydrogen produced and the possibility of catalyst poisoning due to sulfur pollutants in wastewater does not make it the best candidate for HER in MECs for large applications [18]. At present, there are a limited number of studies that have focused on characterizing catalysts operating in neutral conditions required for MEC [19], [20], [21], [22]. Additionally, few platinum group metal-free (PGM-free) catalysts have been reported as HER catalysts in MEC [19], [20], [21], [22], [23]. Among them, molybdenum sulfide (MoS_2_) is the most studied [20]. It has been shown that the performances of MoS_2_ as HER catalyst are quite good also in acidic and alkaline media [24], [25]. However, although Mo is considered to be a PGM-free catalyst, it is not an earth abundant metal and the cost of implementing Mo-based catalysts in large-scale MEC applications is not feasible. Other PGM-free catalyst based on Ni [19], [21], stainless steel (SS) [22] and activated carbon on a stainless steel mesh [23] have been reported as alternatives to Pt as HER catalysts in neutral media with promising results; however, the corresponding overpotentials were still high relative to Pt. PGM-free catalysts based on iron have been recently shown to be promising in acidic and alkaline media [26], [27] for H_2_ evolution. However, to the best of our knowledge, PGM-free catalysts based on Fe-N-C materials have not been yet implemented or reported as HER catalysts in neutral media.

Fe-N-C catalysts have been extensively studied for oxygen reduction reaction (ORR) in acidic [28], [29], [30], [31], neutral [32], [33], [34], [35] and alkaline conditions [36], [37], [38], [39]. The materials were found to be extremely active in all pH conditions and performance was better than Pt in alkaline media [40]. The influence of morphology, surface chemistry and ORR of Fe-N-C materials synthesized by adopting the Sacrificial Support Method (SSM) was systematically investigated [41], [42], [43], [44], [45]. It was analytically shown that the microporous structure of materials will improve ORR activity in neutral and high pH because of the accessibility to active sites and removal of products of reaction [41], [42], [43], [44], [45]. Since the removal and capture of H_2_ produced in real applications is quite challenging, the Sacrificial Support Method was selected for preparation of the Fe-N-C catalysts for enhancing H_2_ degassing.

Another main challenge concerning MECs is that energy has to be supplied to the bioelectrochemical system in order to create the conditions for the HER and production of H_2_. Therefore, the system has a negative energy balance. This can be alleviated or overcome by: i) utilization of low cost and efficient catalytic materials that decrease the energy consumed [23]; ii) the hybridization of an MFC with an MEC, which has been also been successfully reported in literature [46], [47]. In the latter configuration, the external voltage supply for HER was given by the voltage/current generated by the working MFCs, yielding a null energy balance and the production of hydrogen [46], [47]. Since then, several strategies have been adopted to overcome the configuration problem of connecting several hydraulically separated MFCs in series to increase the available voltage/current [48], [49], [50], [51]. It was previously demonstrated that the connection in series of MFCs allowed to boost up the voltage, necessary to power practical application, and to reach much higher power produced [50], [51], [52], [53], [54], [55]. Practical applications of MFCs smartly connected in series have been recently shown [50], [51], [52], [53], [54], [55].

It has been shown previously that current and voltage can also be boosted further by coupling MFCs with external supercapacitors [48], [49], [50], [51], [52], [53], [54], [55]. Although commercial external supercapacitors are suitable for certain applications, some issues remain because of the long recharge time required for devices or sensors [48], [49], [50], [51], [52], [53], [54], [55]. Recently, Santoro et al. shown that the integration of the MFC electrodes as an internal supercapacitor in a supercapacitive microbial fuel cell (SC-MFC) [56], [57], [58] is a new approach to increase the current pulses and it could be used to simplify the combined system MFC-MEC system for energy neutral H_2_ production.

The presence and absence of O_2_ creates a double and opposite anaerobic/aerobic environment inside the single chamber MFC. In fact, bacteria on the anode consume the oxygen guaranteeing strictly anaerobic conditions in proximity of the electrode. These specific conditions and oxidation of acetate substrate push the anode towards low potentials values of ≈−0.5 V (vs Ag/AgCl at pH 7). In contrary, O_2_ is needed and used as oxidant. Oxygen reduction reaction takes place at the cathode. Consequently, the cathode electrode is designed to provide oxidant to the catalytic sites. Therefore, air-breathing cathodes are designed to have constant presence of oxygen and this pushes the potentials towards high values. Different environment conditions lead to the self-polarization of the electrodes with the carbon surface at the anode electrostatically charged negatively and that at the cathode charged positively. Once the electrodes are charged, the excess of charge is balanced by the counter ions from the bulk solution forming a double layer at each surface electrode/electrolyte interface. In this case, the MFC electrodes store electrostatically charges at OCV (**V_max,OC_**) behaving like a charged electrochemical double layer capacitor (EDLC).

The electrodes are then discharged through an electrostatic process with: i) surface charges neutralization and ii) ions released into the electrolyte. The electrostatically stored energy is then released through short but high galvanostatic discharges pulses (GLV) with the consequent production of high power output. After discharge, the MFC is left in rest conditions (OCV) and the electrode equilibrium potential is self-recovered. The electrodes are then self-polarized again to the anodic and cathodic equilibrium potentials and the double layer is self-regenerated. The internal EDLC is then fully recharged and conditions, previous the discharge pulse, are re-established. In those conditions, the MFC electrodes work as a self-powered, self-recharged supercapacitor that is able to generate pulsed current/power [56], [57], [58]. It has been shown that intermittent load implementation enhances the output of a microbial fuel cell [59], [60] and SC-MFC operation follows this direction and operating strategy.

Herein, we report for the first time a system that produces H_2_ by a smart series combination of SC-MFCs and without any additional power supply. It utilizes Fe-based PGM-free HER catalysts. The power pulses of four SC-MFCs single cells and series connected (SC-MFC-SERIES) were evaluated. The SC-MFC-SERIES connection was used to drive the potential of an additional HER electrode (Ad_HER_) to the values required for H_2_ production. Different catalyst materials were tested for the Ad_HER_ including Pt and two novel PGM-free catalysts based on iron. Fe-Aminoantipyrine (Fe-AAPyr) and Fe-Mebendazole (Fe-MBZ) are here presented as novel and alternative low cost catalysts compared to Pt for HER. By combining the improved operational setup (the series connection of SC-MFCs) and the utilization of low-cost Ad_HER_ electrodes, the simultaneous production of H_2_ and current/power pulses were shown.

## 2 Experimental Methods and Materials

Four identical single chamber MFCs with a 125 mL volume were used with an immersed brush anode and PGM-free air-breathing cathode.

### 2.1 Materials Description

#### 2.1.1 Anode Electrode

The anode electrodes were commercial carbon brush (Millirose, USA) with diameter of 3 cm and height of 3 cm. It should be noted that the experiments were conducted with the anodes already well-colonized by electroactive bacteria in MFCs running for at least 6 months [32]. The anode was fully immersed in the solution throughout all experiments.

#### 2.1.2 Cathode Electrode

The air-breathing cathode was prepared using a mixture of activated carbon (AC, SX Ultra, USA), carbon black (CB, Alfa Aesar), polytetrafluoroethylene (PTFE, 60 wt% emulsion, Sigma Aldrich) and iron-aminoantipyrine (Fe-AAPyr). The iron-aminoantipyrine (Fe-AAPyr) catalyst was synthesized using the sacrificial support method (SSM) previously presented [61], [62], [63]. Briefly, AC, CB and PTFE were mixed in a grinder with weight percentages of 70, 10 and 20%, respectively. After 5 minutes of grinding, the mixture was applied to a stainless steel mesh (McMaster, USA) current collector using a metallic die, and Fe-AAPyr was added and mixed with the existing powder. The electrode was pressed at 2 mT (metric tons) for 5 minutes with a hydraulic die (Carver, USA). AC, CB and PTFE loading was 40 ± 2 mg cm^−2^ and the Fe-AAPyr loading was 1.5 ± 0.1 mg cm^−2^. The cathodes had a geometric area of 2.9 cm^2^ exposed to the solution. New cathodes were utilized during all experiments.

#### 2.1.3 Additional electrode for hydrogen evolution reaction (Ad_HER_)

Three different catalysts were used as the additional electrode for hydrogen evolution reaction (AdE_HER_): Pt as the control, and Fe-AAPyr and Fe-MBZ as new PGM-free catalysts for HER in neutral media. AdE_HER_ electrodes had a geometric area of 2.3 × 2.3 cm (5.3 cm^2^). Toray carbon paper was used as current collector for the HER electrodes. The catalyst inks were prepared following the procedure described previously [63]. Briefly, Pt, Fe-AAPyr or Fe-MBZ (120 mg each) were mixed with Nafion^®^ (45 wt.%) and isopropanol and then sonicated for at least 1 hour. An air brush was used to spray the ink directly onto the carbon paper. The carbon paper was set up on a hot plate (T = 60 °C) to evaporate the isopropanol and quickly dry the electrode. Fe-AAPyr and Fe-MBZ loading was 5 ± 0.5 mg cm^−2^, while Pt loading was 0.5 ± 0.05 mg cm^−2^. The cathode was connected to a plastic-covered copper wire and the contact was glued using an epoxy resin to avoid exposure to the solution.

### 2.2 Microbial fuel cell and microbial electrolysis cell configuration and operation

**Fig. 1:**
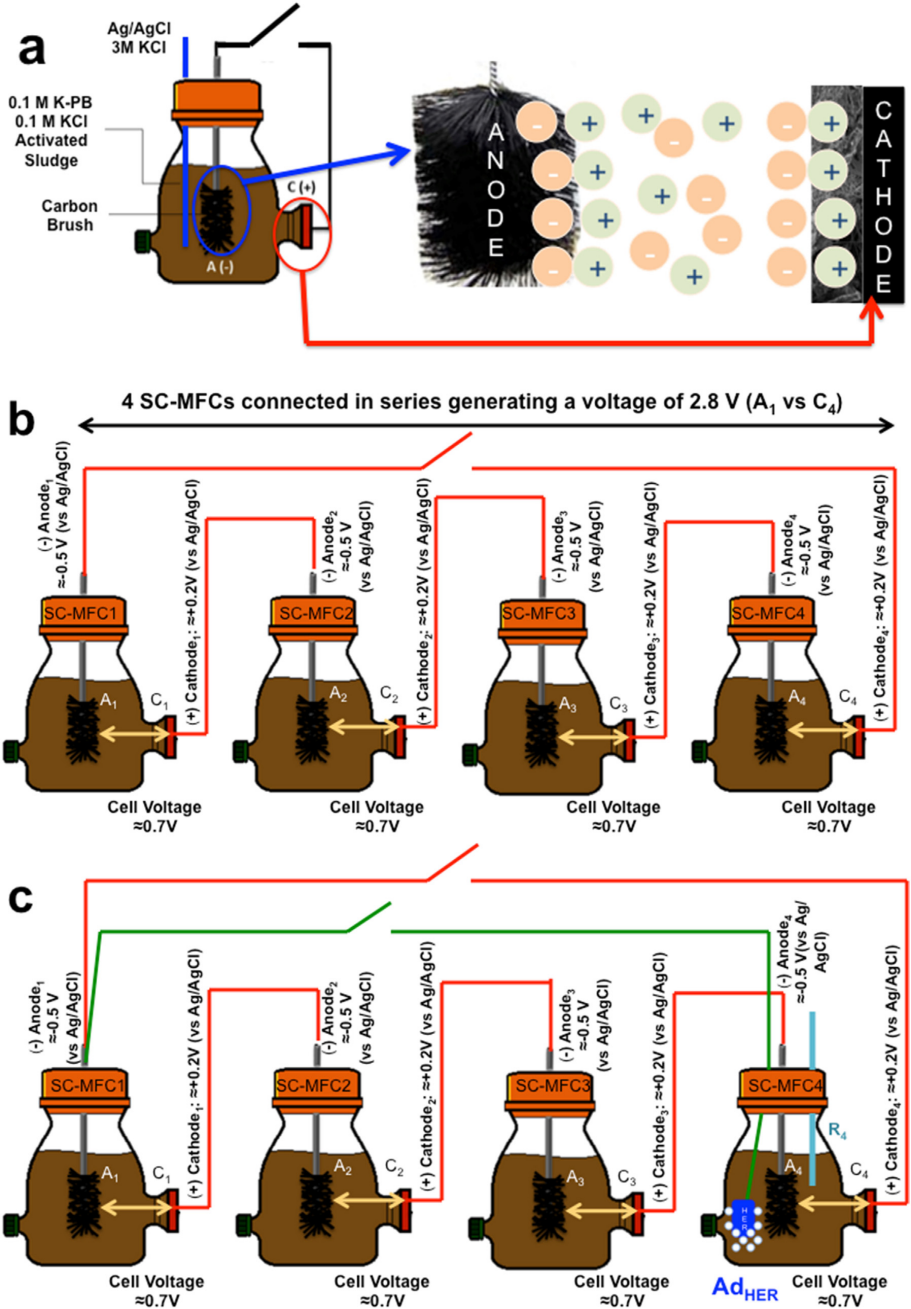
Schematic of a supercapacitive microbial fuel cell with illustration of EDLC formed on the electrodes (a). Schematic of electrochemical reactor consisting of the four SC-MFCs-SERIES (b) in which A is the anode, C is the cathode, R is the reference electrode and the numbers represent the number of the cells. Schematic of the four SC-MFCs-SERIES with the Ad_HER_ in MFC4 for hydrogen evolution (c). A_n_ and C_n_ electrode potentials refer to a nominal Ag/AgCl reference electrode eventually placed in each cell n (not shown).

The electrochemical reactor was filled with a mixture of 50% (v/v) 0.1 M potassium phosphate buffered saline (K-PBS) and 0.1 M of potassium chloride (KCl) solution, and 50% (v/v) activated sludge from the Albuquerque water treatment plant (Albuquerque Southeast Water Reclamation Facility, New Mexico, USA). Acetate in concentration of 3 g L^−1^ (36.5 mM) was used as the carbon source for the electroactive bacteria. All experiments were run with pre-colonized anodes and new sterile cathodes. The four SC-MFCs were characterized separately (Fig. 1a) and then connected in series to increase the operating voltage (Fig. 1b). The four SC-MFCs connected in series were then named SC-MFC-SERIES (Fig. 1b). The series connection was possible because each MFC was hydraulically unique and they did not share the same electrolyte [51], [52].

The SC-MFC-SERIES reactors were then adapted to the Ad_HER_ studies to evaluate H_2_ production. In this case, an Ad_HER_ electrode of appropriate material was added to the last SC-MFC (MFC_4_) of the SC-MFC-SERIES and electrically connected to the anode of first SC-MFC (A_1_) in the SC-MFC-SERIES (Fig. 1c). The pulse timing for opening and closing the circuit between the anode of MFC1 (A_1_) and the Ad_HER_ in MFC4 was varied to evaluate the energy recovery and H_2_ production activities under different pulse-lengths.

### 2.3 Electrochemical measurements

A BioLogic SP-50 potentiostat was used for carrying out electrochemical measurements. Galvanostatic (GLV) discharge curves were performed at different currents (**i_pulse_**) with discharge time (**t_pulse_**) of 2 s and 10 ms. The four SC-MFCs were tested separately using a three-electrode set up with a Ag/AgCl reference electrode (3 M KCl, +210 mV vs SHE) inserted into the solution, the anode electrode as the counter, and the cathode electrode as the working electrode.

**Fig. 2:**
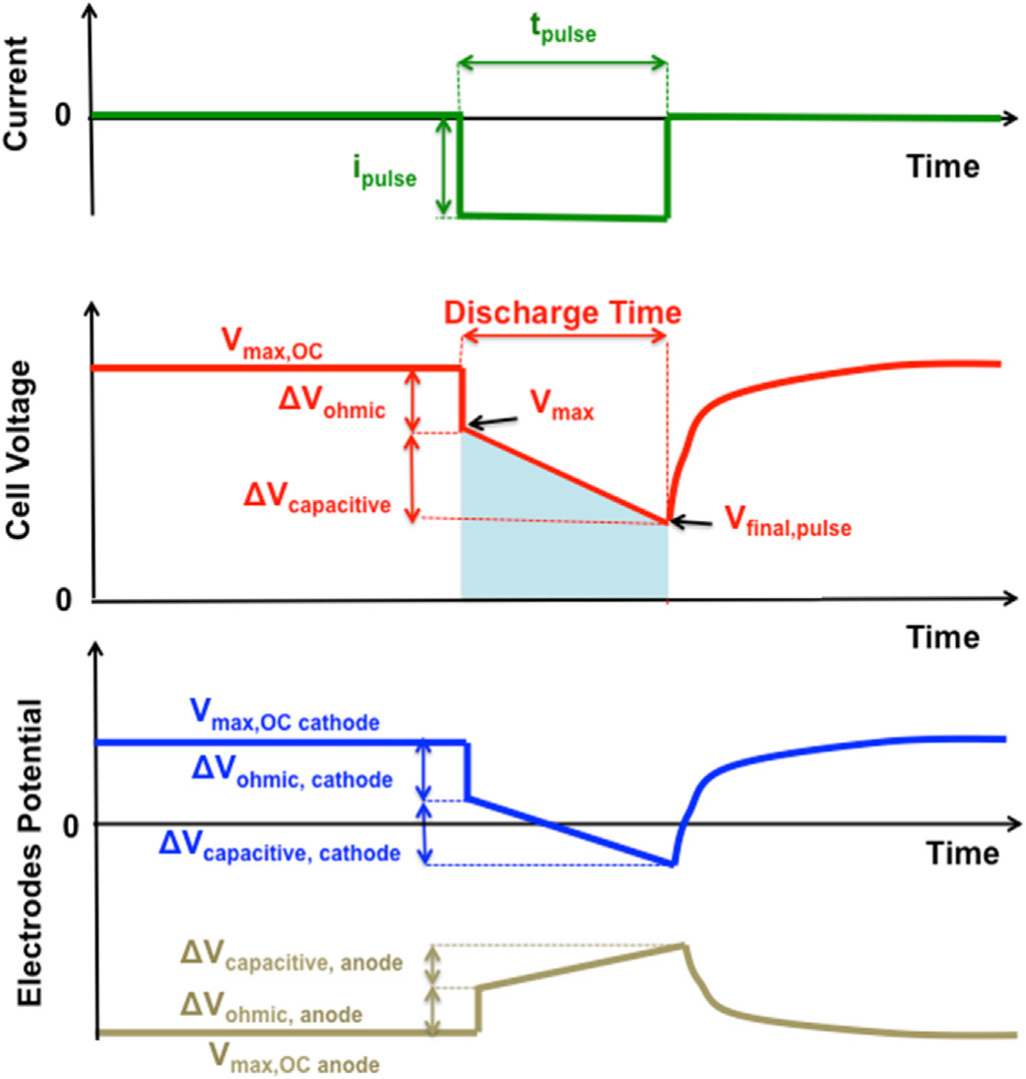
Schematics of galvanostatic discharge curves.

Each SC-MFC was kept under rest conditions (Open Circuit Voltage, OCV or **V_max,OC_**) until stable voltage (±1 mV) was reached and a specific GLV discharge was run for a given time (Fig. 2). After the discharge, the SC-MFC were allowed to recover to the initial **V_max,OC_** value and equilibrium conditions were restored, meaning the SC-MFC was self-recharged.

Following discharge pulse application, the voltage dropped vertically to a lower value (**V_max_**) – that is defined as the practical miximum value of the voltage at which energy and power can be obtained. The ohmic losses (**ΔV_ohmic_**) were quantified as the difference between **V_max,OC_** and **V_max_,** as showed by Eq. (1):

(1) $$\Delta {\text{V}}_{\text{ohmic}}={\text{V}}_{\text{max},\text{OC}}-{\text{V}}_{\text{max}}$$

As a consequence, the equivalent series resistance of each cell (ESR) can be calculated as the ratio between the ohmic losses (**ΔV_ohmic_**) and the constant current applied during the pulse (**i_pulse_**) as showed by Eq. (2):

(2) $$\text{ESR}=\frac{\Delta {\text{V}}_{\text{ohmic}}}{{\text{i}}_{\text{pulse}}}$$

Rearranging Eqs. (1) and Eq. (2), it is possible to express V_max_ differently, accordingly to Eq. (3):

(3) $$V_{max}=V_{max,OC}-\Delta V_{ohmic}=V_{max,OC}-\left(ESR\times i_{pulse}\right)$$

The utilization of the three-electrodes technique allowed us to monitor the electrode potential trends during discharge. After the initial ohmic drop (**ΔV_ohmic_**), the cell voltage linearly decreased over time during the electrostatic discharge (**ΔV_capacitive_**) of the polarized carbon surfaces (anode and cathode). Given that the kinetics of ORR are much slower than the electrostatic process, at high current pulses and short times, the overall SC-MFC response was mainly governed by the electrostatic, capacitive GLV discharge. The ratio between the current pulse (i_pulse_) and the voltage discharge slope (**s**, dV/dt) during t_pulse_ corresponds to the capacitance of the SC-MFC as showed by Eq. (4):

(4) $${\text{C}}_{\text{cell}}=\frac{{\text{i}}_{\text{pulse}}}{\frac{\text{dV}}{\text{dt}}}=\frac{i_{pulse}}{s}$$

In order to have high energy and power, **ΔV_capacitive_** should be minimized and **C_cell_** maximized. The cell capacitance is related to the anode capacitance (C_A_) and cathode capacitance (C_C_) by the following equation:

(5) $${\text{C}}_{\text{cell}}={\left(\frac{1}{{\text{C}}_{\text{A}}}+\frac{1}{{\text{C}}_{\text{C}}}\right)}^{-1}$$

Power curves were constructed considering the maximum power output (**P_max_**) and the power obtained after pulses (**P_pulse_**) of 2 s and 10 ms. Particularly, **P_max_** was calculated by multiplying **V_max_** and the applied current pulse (**i_pulse_**).

(6) $$P_{\max}=V_{\max}\times i_{pulse}=(V_{max,OC}-\left(ESR\times i_{pulse}\right))\times i_{pulse}$$

The energy obtained after a pulse **(E_pulse_**) was calculated as the area under the discharge curve between **V_max_** and **V_final,pulse_** according to Eq. (7):

(7) $${\text{E}}_{\text{pulse}}={\text{i}}_{\text{pulse}}\int_{0}^{\text{t}}\text{V}\;\text{dt}$$

Consequently, the power under the GLV pulse (**P_pulse_**) was calculated as the ratio between the energy produced during a pulse (**E_pulse_**) and **t_pulse_** according to Eq. (8):

(8) $${\text{P}}_{\text{pulse}}=\frac{{\text{E}}_{\text{pulse}}}{{\text{t}}_{\text{pulse}}}$$

**P_max_** and **P_pulse_** were also normalized to the cathode area (2.9 cm^2^) and to the volume of the reactor (125 mL) to establish surface area and volumetric power densities for **P_max_** and **P_pulse_**, respectively.

The four SC-MFCs were then connected in series (SC-MFC-SERIES) and galvanostatically (GLV) discharged after the SC-MFC-SERIES reached a stable voltage under open-circuit conditions. The GLV discharges were conducted with **t_pulse_** of 2 s and 10 ms at a given current (i_pulse_). The data recorded from the SC-MFC-SERIES system were used in the equations above to calculate the maximum energy and power available from the system. However, the three electrode technique was not possible since the SC-MFC-SERIES did not share the same electrolyte and consequently, 2-electrode discharges were performed using the cathode from MFC4 (C_4_) as the positive electrode and the anode from MFC1 (A_1_) as the negative electrode (Fig. 1b). **P_max_** and **P_pulse_** curves were then calculated as previously described.

After the SC-MFC-SERIES system was characterized for power and energy, the additional electrode for hydrogen evolution reaction (Ad_HER_) was inserted in MFC_4_ and short-circuited to A_1_ as shown in Fig. 1c. This connection drove the Ad_HER_ potential to the low values necessary for generating H_2_. If redox processes are not taking place (e.g. in presence of non-aqueous, aprotic media) this connection should drive the Ad_HER_ to the potential corresponding to the sum of all other anode electrodes (A_1_ + A_2_ + A_3_ + A_4_). Practically, in an aqueous environment the lowest feasible potential for Ad_HER_ is set by the potential at which the electrocatalytic hydrogen evolution occurs. The potential of the Ad_HER_ electrode vs Ag/AgCl (R_4_) and the potential difference between A_1_ and C_4_ of the SC-MFC-SERIES (i.e. the SC-MFC-SERIES voltage) were monitored over time. The system reached the operational equilibrium (stable Ad_HER_ potential and stable SC-MFC-SERIES voltage) after roughly 1 hour. After the stabilization, the following electrochemical performances were monitored simultaneously.

GLV discharges were then carried out with simultaneous production of H_2_. Particularly, two tests were simultaneously performed: i) the potential of the Ad_HER_ electrode vs Ag/AgCl (R_4_) was monitored over time, and ii) galvanostatic discharges were performed using C_4_ as positive electrode and A_1_ as negative electrode while the HER was taking place. These experiments were feasible by using a two-channel potentiostat.

### 2.4 Hydrogen Evolution Reaction Measurements

#### 2.4.1 HER measurements

The hydrogen evolution reaction of the Pt and PGM-free catalysts were initially run in neutral media for studying the electroactivity of those catalysts towards HER. The parameters of interests were: i) onset potential; ii) overpotential (in respect to platinum); and iii) potential at *i* = 20 mA cm^−2^. The HER was initially measured separately in a hermetically closed Pyrex glass chamber (volume 100 mL) containing potassium phosphate buffer (K-PB) solution (0.1 M with 0.1 M KCl) at pH 7.5 (Fig. S1). The HER was measured using a three-electrode setup with the HER electrode as the working, a platinum mesh as the counter and Ag/AgCl (3 M KCl, +210 mV vs SHE) as the reference. The working electrodes were Pt, Fe-AAPyr, Fe-MBZ and carbon paper (CP), where Pt and CP were used as controls. Each HER electrode was submerged in the electrolyte solution overnight to increase the electrode wettability and avoid any oxygen molecules adsorbed on the surface. N_2_ was purged vigorously for at least 30 minutes prior to starting the experiments. Linear Sweep Voltammetry (LSV) between open circuit potential (OCP) and −2 V vs Ag/AgCl was run at a scan rate of 1 mV s^−1^ in separate triplicates electrodes.

After the HER tests in neutral media, the electrodes were used as Ad_HER_ as showed in Fig. 1c. H_2_ production from the Ad_HER_ during the operations was estimated using the Faraday law. Particularly, the molar of H_2_ evolution rate was determined using the following Eq. (9):

(9) $$\dot{n}=\frac{2\times {\text{i}}_{\text{HER}}}{\text{F}}$$

where $\dot{n}$ is the molar rate (mol s^−1^) of hydrogen production, 2 is the number moles of electrons necessary to generate 1 mole of H_2_, *i_HER_* is the HER current (A) and F is the Faraday constant 96485 (C mol^−1^).

*i_HER_* was evaluated by separate chronoamperometric experiments run in single SC-MFC cells using a three-electrode configuration that simulates Ad_HER_ operation when driven by the SC-MFC-SERIES system. Ad_HER_ was used as working electrode, the counter was an anode (carbon brush) and an Ag/AgCl was used as reference (Fig. S2). During the chronoamperometric measurements the Ad_HER_ potential was set at the value recorded under operation in the SC-MFC-SERIES system, i.e. after the short-circuit of Ad_HER_ with A_1_ in MFC_4_. The H_2_ production was showed as mol day^−1^ cm^−2^ in which mol day^−1^ is the rate evolution of H_2_ per the geometric area of the electrode (5.9 cm^2^). Moreover, the H_2_ was reported as volume produced (L day^−1^). H_2_ was considered as a perfect gas and the equation PV = nRT was used to estimate the volume occupied by 1 mol of H_2_ at 1600 AMSL with an atmospheric pressure calculated as 0.83 atm and a room temperature of 22 °C (295°K). The volume of H_2_ in those operating conditions is 29 L mol^−1^.

#### 2.4.2 Hydrogen production using Gas Chromatography

The Faraday law allowed estimation of the H_2_ produced at the AdE_HER_. To ensure that H_2_ was produced during the experiments, parallel trials on the four SC-MFC-SERIES system with the Ad_HER_ were executed with the Fe-AAPyr Ad_HER_ catalyst and headspace samples were extracted for gas chromatography (GC). The SC-MFC-SERIES system was run overnight (15 hours) and then H_2_ production was quantitatively measured using an Agilent 6890N gas chromatograph with ChemStation software Rev. B.02.01-SR1 (260) and a Varian Chrompack Capillary column, CP-Molsieve 5 Å 10 m x 0.53 mm × 50 μm (PN CP7573). A 0.25 mL manual injection of reactor headspace was run on a 1 min method using a split mode inlet at 120 °C with N_2_ carrier gas (5.84 psi, 115 mL min^−1^), column flow of N_2_ (5.84 psi, 10.2 mL min^−1^, 115 cm sec^−1^), oven temperature of 80 °C, and a thermal conductivity detector at 120 °C (reference flow at 10 mL min^−1^, makeup flow N_2_ at 5 mL min^−1^). A standard calibration curve was prepared using 5% H_2_: 95% N_2_ (v/v) at different injection volumes, producing a sharp H_2_ peak at ∼0.35 mins and a broad background peak at ∼0.42 mins.

## 3 Results and Discussion

### 3.1 Supercapacitive microbial fuel cell performances

**Fig. 3:**
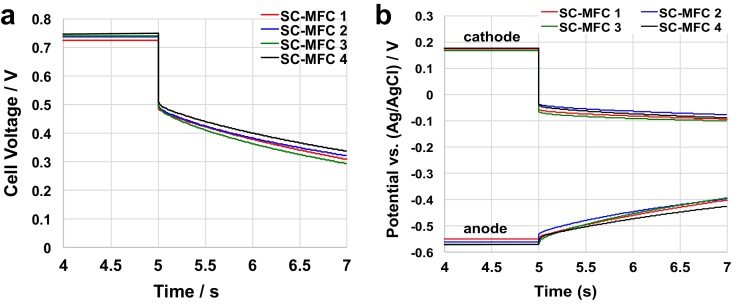
Cell voltage (a) and electrode potential (b) profiles of four different SC-MFCs under 5 s rest and 2 s pulses at 3 mA.

Four SC-MFCs were tested separately using GLV discharges with **t_pulse_** of 2 s and **i_pulse_** of 3 mA. The cell voltage and the anode/cathode potential trends were recorded and shown in Fig. 3a and b, respectively. Those specific values of **t_pulse_** and **i_pulse_** were adopted in order to compare to previous reported data on similar operating conditions [56], [57], [58], [64]. The behaviors of the four separate cells were very similar and comparable to each other indicating reproducibility of the materials utilized and the process. The SC-MFCs exhibited a **V_max,OC_** of 740 ± 10 mV (Fig. 3a) which resulted from a contribution of 175 ± 5 mV (vs Ag/AgCl 3 M KCl) of the cathode and −570 ± 11 mV (vs Ag/AgCl 3 M KCl) of the anode (Fig. 3b). The overall discharge with an **i_pulse_** of 3 mA showed an ohmic drop of 230 ± 6 mV corresponding to an ESR of 77 ± 2 Ω. This was mainly due to the cathode (Fig. 3b) ohmic drop that contributed roughly 95% to **ΔV_ohmic_**. These results are in agreement with previously presented data [56], [57], [58]. Moreover, the capacitance of the cell (C_cell_) can be extrapolated (Eq. (5)) by the curves shown in Fig. 3a. Overall C_cell_ was 16 ± 1.1 mF, C_cathode_ was 71 ± 9 mF and C_anode_ was 20 ± 2 mF.

### 3.2 SC-MFC power curves

**Fig. 4:**
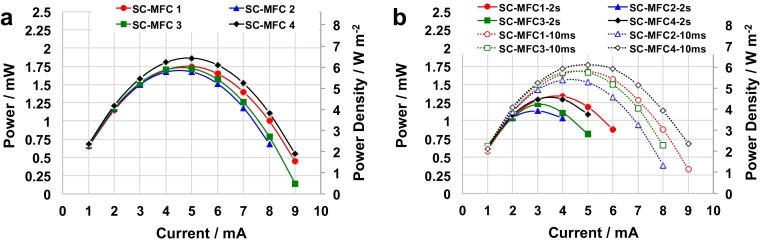
P_max_ vs I_max_ plots for SC-MFC (a). P_pulse_ vs i_pulse_ plots for SC-MFC for pulse time of 10 ms and 2 s (b).

Power curves were then generated with the intent of evaluating the performances of the four separate SC-MFCs. Power curves (**P_max_**) were built from the discharge curves at 3 mA considering **V_max,OC_** of 737 ± 10 mV and **ESR** of 77 ± 2 Ω (Fig. 4a). **P_max_** of 1.75 mW (at 5 mA), 1.68 mW (at 4 mA), 1.72 mW (at 5 mA) and 1.87 mW (at 5 mA) was detected for SC-MFC_1_, SC-MFC_2_, SC-MFC_3_ and SC-MFC_4_, respectively (Fig. 4a). **P_max_** was also expressed as a function of the cathode area and the volume of the reactor with SC-MFC_1_ producing 6 W m^−2^ (14 W m^−3^), 5.8 W m^−2^ (13.4 W m^−3^) for SC-MFC_2_, 5.9 W m^−2^ (13.8 W m^−3^) for SC-MFC_3_ and 6.4 W m^−2^ (14.9 W m^−3^) SC-MFC_4_. A maximum current of 8 mA was measured for SC-MFC_2_, while SC-MFC_1_, SC-MFC_3_ and SC-MFC_4_ were able to reach 9 mA as maximum i_pulse_.

**P_pulse_** of 10 ms was slightly lower than **P_max_** varying between 1.6 mW (5.3 W m^−2^, 12.4 W m^−3^, SC-MFC_2_) and 1.8 mW (6.1 W m^−2^, 14.2 W m^−3^, SC-MFC_4_) (Fig. 4b). Lower **P_pulse_** was then achieved with a t_pulse_ of 2 s for all SC-MFCs. In fact, **P_pulse_** (2 s) varied between a minimum of 1.1 mW for SC-MFC_2_ (3.9 W m^−2^, 9 W m^−3^) and a maximum of 1.3 mW for SC-MFC_1_ (4.6 W m^−2^, 10.6 W m^−3^) (Fig. 4b). Performance differences of only 10% were detected among the four SC-MFCs, indicating good reproducibility. Consequently, the utilization of SC-MFC follows the direction of enhancing power output of a MFC system. Those performances were similar to the one previously presented in SC-MFCs working with similar electrolyte and same electrode materials [56], [57], [58]. Power pulses obtained are much higher than continuous power produced in a single chamber MFC [56], [57], [58].

### 3.3 SC-MFC-SERIES System Performance

**Fig. 5:**
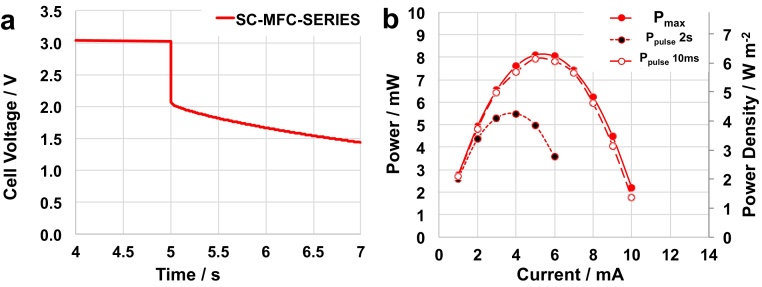
Cell voltage (a) profiles of SC-MFC-SERIES under 5 s rest and 2 s pulse at 3 mA (a). P_max_ vs I and P_pulse_ vs i_pulse_ plots (t_pulse_ of 10 ms and 2 s) for SC-MFC-SERIES (b).

The connection in series of the four SC-MFCs (SC-MFC-SERIES) provided a voltage, i.e. the the potential difference between A_1_ and C_4,_ that was approximately four-folds the value of each SC-MFC. The SC-MFC-SERIES system had an OCV of 3025 mV. The connection in series was possible due to the hydraulic disconnection of the different reactors. To evaluate the system, cell discharges at 3 mA were executed and the data are presented in Fig. 5a. The ESR of SC-MFC-SERIES was ≈300 Ω, equivalent to an average of ≈75 Ω for each SC-MFC. This average ESR was similar to the ones measured for each single reactor. Power curves were then measured for SC-MFC-SERIES (Fig. 5b). SC-MFC-SERIES had a P_max_ of 8.1 mW (6.2 W m^−2^, 14.4 W m^−3^, at 5 mA), P_pulse_ (t_pulse_ 10 ms) of 7.9 mW (6 W m^−2^, 14.1 W m^−3^, at 4 mA) and P_pulse_ (t_pulse_ 2 s) of 5.5 mW (4.3 W m^−2^, 9.6 W m^−3^, at 4 mA). Here for the first time, it is shown the successful improvements in power/current/voltage output of SC-MFC connected in series.

### 3.4 HER on Pt, Fe-AAPyr and Fe-MBZ electrodes in neutral media

**Fig. 6:**
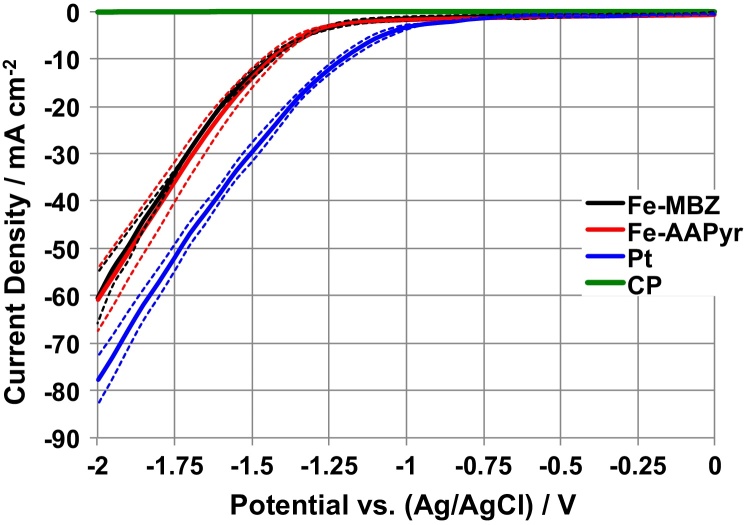
Linear sweep voltammetry at Fe-MBZ, Fe-AAPyr, Pt and CP electrodes at neutral pH in N_2_-saturated electrolyte between 0 and −2 V vs Ag/AgCl at 1 mVs^−1^.

Hydrogen evolution reaction at Fe-MBZ, Fe-AAPyr, Pt and CP electrodes was investigated by linear sweep voltammetry in neutral pH (K-PB solution, pH = 7.5, T = 25 °C) and N_2_-saturated electrolyte between 0 and −2 V (vs Ag/AgCl) and 1 mV s^−1^ using a conventional three-electrode as showed in Fig. 6. Separate experiments were run to determine of HER parameters of interest such as onset potential, overpotential (in respect to platinum), and potential at *i* = 20 mA cm^−2^ for the different HER catalysts (Fig. 6). As we discussed earlier, the neutral conditions are not considered favorable for paired reactions (ORR/OER and HOR/HER) due to the fact that protons and hydroxyls are the main reagents in those processes. It is clear that the concentration of both H^+^ and OH^−^ is the lowest at pH values approaching 7.

Fig. 6 shows that Pt has the lowest overpotential related to the materials tested. Onset potential for Pt was −0.75 V (vs Ag/AgCl) and at *i* = 20 mA cm^−2^ the potential was −1.35 V (vs Ag/AgCl). PGM-free electrocatalysts, as expected, have a higher onset in the range of −1/-1.1 V (vs Ag/AgCl) with a potential of 1.52 V (vs Ag/AgCl) at *i* = 20 mA cm^−2^. According to the Faraday law, the H_2_ produced at *i* = 20 mA cm^−2^ corresponds to 18 mM day^−1^ cm^−2^ or 5.5 L day^−1^. In general, the overpotential of PGM-free catalysts was ∼250 mV higher than platinum, indicating that Fe-based materials are promising catalysts for hydrogen production in MECs. Overpotentials are much lower compared to Ni and SS previously presented as substitute of Pt in neutral media [65], [66].

### 3.5 SC-MFC-SERIES voltage and Ad_HER_ potential

**Fig. 7:**
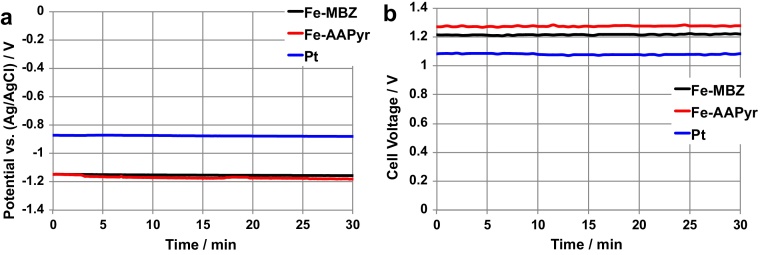
Potential of the different HER electrodes (Ad_HER_) in MFC-4 after 5000 second stabilization (a). SC-MFC-SERIES voltage during discharges while simultaneously producing hydrogen (b).

After the study of HER electrodes separately in neutral media electrolytes that was carried out to investigate their activity (section 3.4), the electrodes were used as Ad_HER_ electrode and connected as in Fig. 1c. The Ad_HER_ electrode was added into the anodic chamber of MFC_4_ and short circuited with A_1_ (Fig. 1c). The Ad_HER_ potential was driven to low values, reaching a stable value after approximately 60 minutes. The stable Ad_HER_ potential output for Pt, Fe-AAPyr and Fe-MBZ is shown in Fig. 7a over 30 minutes operation. Pt stabilized at −0.88 V (vs Ag/AgCl) while Fe-AAPyr and Fe-MBZ stabilized at lower values of −1.17 V (vs Ag/AgCl) and −1.15 V (vs Ag/AgCl) respectively (Fig. 7a). SC-MFC-SERIES decreased their voltage to lower values recorded in 1.27 V for SC-MFC-SERIES with Fe-AAPyr as Ad_HER_, 1.23 V for SC-MFC-SERIES with Fe-MBZ as Ad_HER_ and 1.12 V for SC-MFC-SERIES with Pt as Ad_HER_ (Fig. 7b).

### 3.6 Hydrogen Production from Ad_HER_

**Fig. 8:**
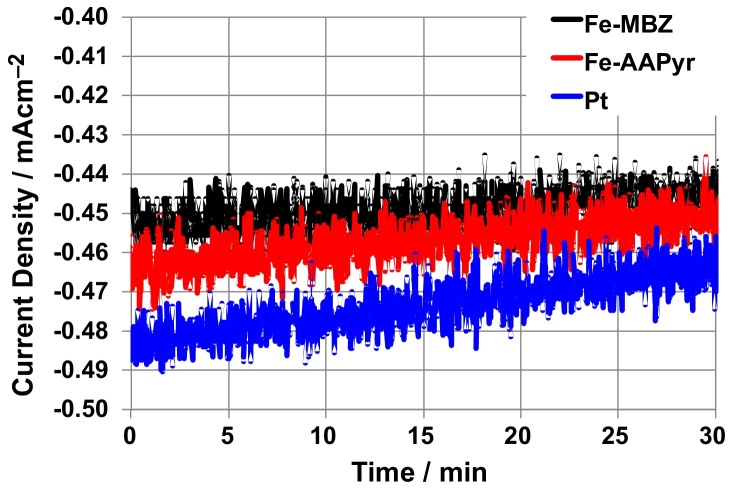
Chronoamperometry plots for Ad_HER_ electrodes with Fe-MBZ, Fe-AAPyr, and Pt at applied potentials of −1.15 V, −1.17 V, and −0.88 V vs Ag/AgCl, respectively.

Chronoamperometry was used to simulate and investigate Ad_HER_ processes at the potentials driven by SC-MFC-SERIES and, specifically, at those reported in from Fig. 7a. For Fe-MBZ, Fe-AAPyr, and Pt the potentials −1.15 V, −1.17 V and −0.88 V vs Ag/AgCl were applied, respectively. The average current values recorded during chronoamperometry after stabilization (roughly 1 hour) were very similar and equal to −0.47 mA cm^−2^ for Pt, −0.46 mA cm^−2^ for Fe-AAPyr and −0.45 mA cm^−2^ for Fe-MBZ (Fig. 8). This current generated was used to estimate the H_2_ production (supposing 100% yield) by Eq. (9) which resulted of 0.86, 0.83, and 0.80 mMday^−1^cm^−2^ with Pt, Fe-AAPyr, and Fe-MBZ catalysts, respectively. In terms of volumetric H_2_ production, the values above correspond to 0.132 L day^−1^ (Pt), 0.127 L day^−1^ (Fe-AAPyr), 0.123 L day^−1^. To confirm the production of H_2_, the system shown in Fig. 1c with Ad_HER_ featuring Fe-AAPyr was run for 15 hours and then the headspace of MFC_4_ was sampled and analyzed using a GC. The measurement confirmed that hydrogen occupied 44% of the gas headspace.

The utilization of the Faraday law may give an overestimation of the hydrogen produced. Likely part of the H_2_ produced is lost due to bacteria uptake and utilization. In fact, differently than the HER processes happening in abiotic conditions, the chamber contains different kind of bacteria which utilize hydrogen directly or through intermediate process in which the latter is involved and consumed. Consequently, we can postulate that not all the H_2_ produced during the HER is successfully recovered and future work will be devoted to improve cell configuration a to minimize H_2_ loss during the HER.

### 3.7 Simultaneous Pulsed Power and HER

**Fig. 9:**
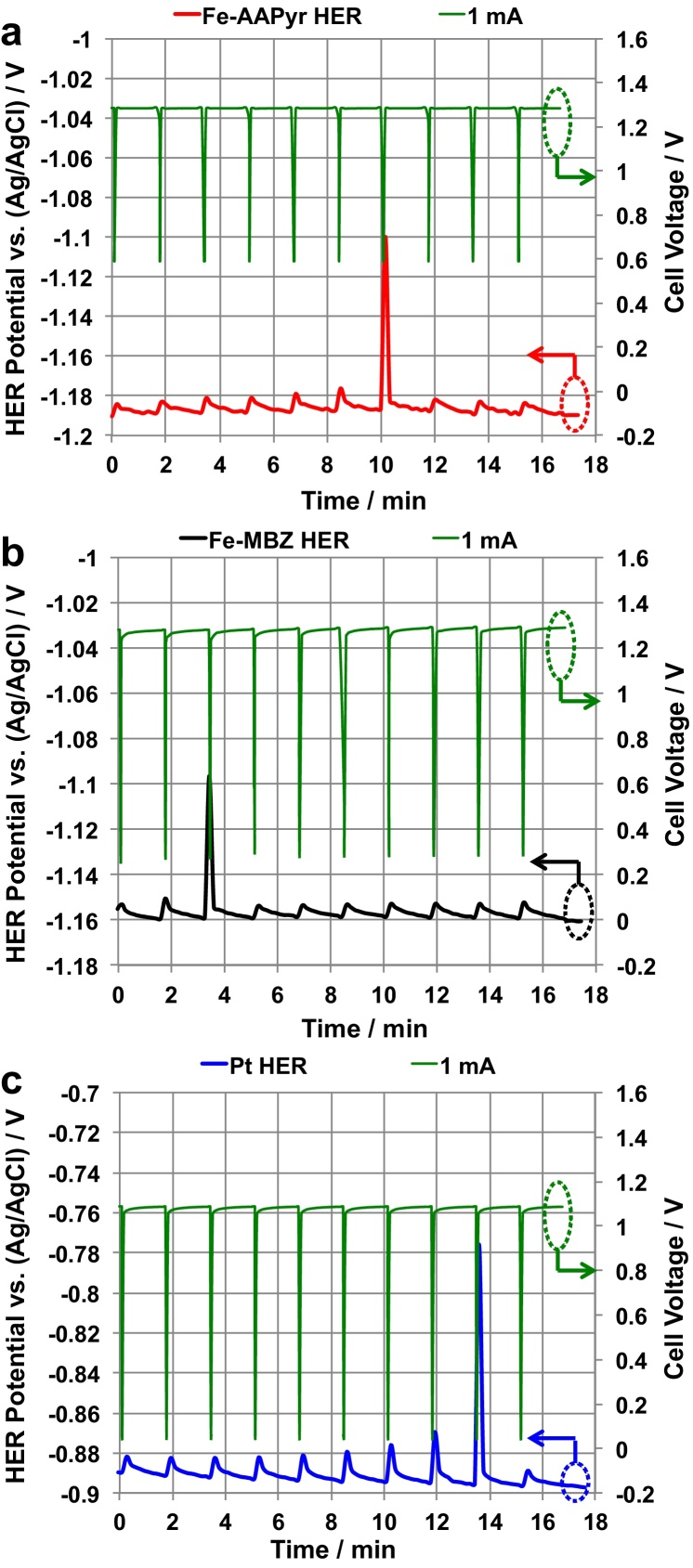
Simultaneous hydrogen evolution (bottom line) and discharge curves (upper line) using Fe-AAPyr (a, red), Fe-MBZ (b, black) and Pt (c, blue) as the Ad_HER_ electrode. The green upper curves are SC-MFC-SERIES voltage profiles. (For interpretation of the references to colour in this figure legend, the reader is referred to the web version of this article.)

After 30 min stabilization of the Ad_HER_ potential (Fig. 7), 10 discharges of the SC-MFC-SERIES were carried out with t_pulse_ of 2 s and rest time of 100 s. The 10 cycles were repeated for discharge currents (i_pulse_) of 1 mA (Fig. 9). The potential of the Ad_HER_ was recorded continuously vs an Ag/AgCl reference electrode placed in the same chamber n.4 (Fig. 1c). Simultaneously, the SC-MFC-SERIES voltage under the GLV discharges/self-recharges was monitored. The SC-MFC-SERIES voltage corresponds to the difference between the C_4_ positive electrode and the A_1_ negative electrode (Fig. 1c).

During the GLV discharges, the HER electrodes slightly increased their potentials. Indeed, the Ad_HER_ follow the A1 electrodes to which are connected and that increase their potential, following the electrostatic depolarization, during the GLV discharge pulses. In any case, the Ad_HER_ potentials always keep well within the range where H_2_ is produced (Fig. 9). Discharges of 1 mA were showed when Fe-AAPyr, Fe-MBZ and Pt were used as the Ad_HER_ catalysts (Fig. 9).

In this configuration, for the first time we show the possibility of producing H_2_ through a smart connection of MFCs utilizing an additional HER. Moreover, for the first time, Fe-AAPyr and Fe-MBZ were used as catalysts for HER in neutral media and had similar HER production rates to Pt, the most commonly used catalysts. However, Fe-AAPyr and Fe-MBZ are better options than Pt due to their lower costs, higher tolerance to pollution, and durability in harsher or more realistic conditions.

## 4 Outlook

The utilization of high surface area and conductive electrode materials allowed to use the MFC electrodes as supercapacitor electrodes under pulsed operational mode. Pulsed power generated was substantially higher than continuous power generation in agreement with previously presented literature [56], [58]. It was previously shown that the intermittent operational mode is quite more effective that continuous mode operation [59], [60]. If the SC-MFCs are not hydraulically connected, a series connection is possible in order to enhance the operating voltage of the system [49], [50], [51]. High voltage is necessary for practical applications [49], [50], [51] and in this specific experimental study, voltage of over 3 V was achieved. In this current work, we showed the possibility of integrating an in-series connection of MFCs working in supercapacitive mode (SC-MFC-SERIES) which are able to generate H_2_ through an additional electrode devoted to the HER. Integration of the HER electrode along with the supercapacitors allowed us to design a co-generative system where the electricity generated can be further utilized for the simultaneous production of H_2_ with no additional external sources operated. This synergistic integration is very promising since the higher combined anodic activity creates current pulses large enough to be delivered to external sources.

Meanwhile, H_2_ produced at the additional electrode can be harnessed for various applications. The hydrogen evolution reaction took place within MFC_4_ with no utilization of external power sources aside from the MFCs themselves, which have a positive electricity production. This way of thinking about energy and waste is quite revolutionary and innovative as it creates a system that is self-sustainable with positive energy output and marketable compounds (H_2_). To the best of our knowledge, the existing literature does not show simultaneous production of current/power high quality pulses and H_2_. Pt is often utilized as a catalyst for the HER, hindering the possibility of large-scale applications due to elevated material costs and the easy inactivation when working in polluted conditions. Here, we introduced novel PGM-free catalysts for HER that work effectively in neutral media and in wastewater conditions with many pollutants. It is the first time that Fe-based catalysts have been used for HER in neutral media, and since these catalysts use commonly available and cheap metals, they are much more suitable for large-scale applications requiring durability. These materials showed higher overpotentials compared to Pt but they are still promising as a substitute and more long-term evaluations of these materials under different operational conditions should be executed to further validate these findings.

## 5 Conclusions

SC-MFCs were studied separately and then were connected in series to increase current/voltage for use in practical applications. Maximum power of the single SC-MFC was 1.8 ± 0.1 mW that is equivalent to 6.1 ± 0.3 W m^−2^ and 14.0 ± 0.7 W m^−3^. Those power output are significantly higher than any presented MFC power output. When four SC-MFCs were operated in series, the voltage increased to over 3 V for the system and the maximum power was 8.1 mW (6.2 W m^−2^, 14.4 W m^−3^). This system with an additional electrode (Ad_HER_) in one of the SC-MFCs was utilized for the simultaneous production of hydrogen and power. Current pulses and H_2_ production were shown to take place simultaneously. Hydrogen evolution reaction catalysts based on Fe (instead of Pt) with relatively low overpotentials were successfully implemented for the first time.
