# Supplementary material for: Co-generation of hydrogen and power/current pulses from supercapacitive MFCs using novel HER iron-based catalysts
Source: Electrochim Acta. 2016 Dec 1;220:672–82. doi: 10.1016/j.electacta.2016.10.154 (PMC5127565; doi:10.1016/j.electacta.2016.10.154)
Supplement: Supplementary file 1 [file mmc1.docx]

**Supporting Information**

**Co-generation of hydrogen and power/current pulses from supercapacitive MFCs using novel HER iron-based catalysts**

*Carlo Santoro^1,a^, **Francesca Soavi^2,a^, Catia Arbizzani^2^, Alexey Serov^1^, Sadia Kabir^1^, Kayla Carpenter^3^, Orianna Bretschger^3^, Plamen Atanassov^1^.

^1^ Department of Chemical and Biological Engineering, Center for Micro-Engineered Materials (CMEM), University of New Mexico, Albuquerque, NM 87131, USA

^2^ Department of Chemistry “Giacomo Ciamician”, Alma Mater Studiorum - Università di Bologna, Via Selmi, 2, 40126 Bologna, Italy.

^3^ J. Craig Venter Institute, 4120 Capricorn Lane, La Jolla, CA 92037, USA

^a^ The two authors contributed equally to the manuscript

* Carlo Santoro, Department of Chemical & Biological Engineering, Center for Micro-Engineered Materials (CMEM), University of New Mexico, Albuquerque, NM 87131, USA. E-mail: santoro@unm.edu

** Francesca Soavi, Department of Chemistry “Giacomo Ciamician”, Alma Mater Studiorum - Università di Bologna, Via Selmi, 2, 40126 Bologna, Italy. E-mail: [francesca.soavi@unibo.it](mailto:francesca.soavi@unibo.it)

**Figure S1.** Three electrode configuration for studying hydrogen evolution reaction of the materials investigated in clean media. LSV was run between 0 V (vs Ag/AgCl) and -2 V (vs Ag/AgCl) at scan rate of 1 mV s^-1^.

**Figure S2.** Three electrode configuration for studying Ad_HER_ hydrogen evolution reaction of the materials investigated in working media. Chronoamperometry was run at the stable potential recorded in Figure 8.a.
